# Long-read sequencing reveals a 4.4 kb tandem repeat region in the mitogenome of *Echinococcus granulosus* (*sensu stricto*) genotype G1

**DOI:** 10.1186/s13071-019-3492-x

**Published:** 2019-05-16

**Authors:** Liina Kinkar, Pasi K. Korhonen, Huimin Cai, Charles G. Gauci, Marshall W. Lightowlers, Urmas Saarma, David J. Jenkins, Jiandong Li, Junhua Li, Neil D. Young, Robin B. Gasser

**Affiliations:** 1 Faculty of Veterinary and Agricultural Sciences, The University of Melbourne, Parkville, VIC Australia; 2 BGI Research, Shenzhen, Guangdong China; 3 Department of Zoology, Institute of Ecology and Earth Sciences, University of Tartu, Tartu, Estonia; 4 School of Animal and Veterinary Sciences, Charles Sturt University, Wagga, Wagga, NSW Australia

**Keywords:** *Echinococcus*, Genotype G1, Complete mitochondrial (mt) genome, Repetitive DNA, PacBio sequencing

## Abstract

**Background:**

*Echinococcus* tapeworms cause a severe helminthic zoonosis called echinococcosis. The genus comprises various species and genotypes, of which *E. granulosus* (*sensu stricto*) represents a significant global public health and socioeconomic burden. Mitochondrial (mt) genomes have provided useful genetic markers to explore the nature and extent of genetic diversity within *Echinococcus* and have underpinned phylogenetic and population structure analyses of this genus. Our recent work indicated a sequence gap (> 1 kb) in the mt genomes of *E. granulosus* genotype G1, which could not be determined by PCR-based Sanger sequencing. The aim of the present study was to define the complete mt genome, irrespective of structural complexities, using a long-read sequencing method.

**Methods:**

We extracted high molecular weight genomic DNA from protoscoleces from a single cyst of *E. granulosus* genotype G1 from a sheep from Australia using a conventional method and sequenced it using PacBio Sequel (long-read) technology, complemented by BGISEQ-500 short-read sequencing. Sequence data obtained were assembled using a recently-developed workflow.

**Results:**

We assembled a complete mt genome sequence of 17,675 bp, which is > 4 kb larger than the complete mt genomes known for *E. granulosus* genotype G1. This assembly includes a previously-elusive tandem repeat region, which is 4417 bp long and consists of ten near-identical 441–445 bp repeat units, each harbouring a 184 bp non-coding region and adjacent regions. We also identified a short non-coding region of 183 bp, which includes an inverted repeat.

**Conclusions:**

We report what we consider to be the first complete mt genome of *E. granulosus* genotype G1 and characterise all repeat regions in this genome. The numbers, sizes, sequences and functions of tandem repeat regions remain to be studied in different isolates of genotype G1 and in other genotypes and species. The discovery of such ‘new’ repeat elements in the mt genome of genotype G1 by PacBio sequencing raises a question about the completeness of some published genomes of taeniid cestodes assembled from conventional or short-read sequence datasets. This study shows that long-read sequencing readily overcomes the challenges of assembling repeat elements to achieve improved genomes.

## Background

Cestodes of the genus *Echinococcus* cause a disease called echinococcosis, which affects humans, and various domestic and wild mammals [1]. *Echinococcus* spp. are distributed worldwide and represent a substantial global public health and socioeconomic burden [2]. Echinococcosis is recognised by the World Health Organization (WHO) as a neglected tropical disease (NTD), requiring a prioritisation of global research and control efforts [3].

Genetically, *Echinococcus* is a diverse cestode group, currently consisting of 10 species [4–7]: *E. multilocularis*, *E. oligarthra*, *E. vogeli*, *E. shiquicus*, *E. granulosus* (*sensu stricto*; genotypes G1 and G3), *E. equinus* (genotype G4), *E. ortleppi* (genotype G5), *E. intermedius* (species name is being debated [6, 8–10]; comprising genotypes G6 and G7), *E. canadensis* (genotypes G8 and G10) and *E. felidis*. These species are distinctly different from one another in their ecology (e.g. infectivity to humans, prevalence, distribution and host ranges) [1]; thus, exploring the extent of genetic variation within the genus *Echinococcus* is central to understanding disease transmission patterns. *Echinococcus granulosus* genotype G1 is recognised as the most wide-spread of all *Echinococcus* taxa, and is, thus, of particular importance [2, 11].

Mitochondrial (mt) genomes have provided useful genetic markers to discover the nature and extent of genetic diversity within *Echinococcus* [7, 12–15], and have underpinned extensive phylogenetic and population structure analyses of this genus over the years (e.g. [16–20]). Published reports show that mt genomes of genotype G1 sequenced using Sanger-, 454- or Illumina-methods are ~ 13,600 bp in length [16, 21, 22]; in addition to 12 protein-encoding genes, 22 tRNAs and 2 rRNAs, the mt genome contains two non-coding regions (NCRs), one of which is between tRNA-Tyr and tRNA-Leu genes (NR1; estimated at 87 bp [16] or 66 bp [21]) and the other between *nad*5 and tRNA-Gly (NR2, estimated at 184 bp; [16, 21]). However, our recent work [23], exploring the “global” genetic structure of *E. granulosus* genotype G1 using near-complete mitogenome sequences of 211 individual samples of this genotype revealed a gap (estimated at > 1 kb) between the 3′-end of the *nad*5 gene and the 5′-end of the *cox*3 gene. In spite of many attempts to PCR-amplify (using a range of different oligonucleotide primer sets designed specifically to the *nad*5 and *cox*3 genes flanking this enigmatic region), we were not able to define this region for any of the 211 genotype G1 isolates investigated using a Sanger-based sequencing approach (cf. [23]). This finding was suggestive of a repetitive, non-coding region of complex structure(s) that is “resistant” to amplification by conventional PCR. This challenge needed to be circumvented using a different approach.

The availability of PacBio sequencing technology [24] and the advent of an automated pipeline [25] for the assembly of long-read sequence data provided an opportunity to overcome this obstacle and to, for the first time, directly define a complete mt genome of *Echinococcus*, in one sweep, irrespective of the nature or structural complexities in intergenic regions. Here, we report what we consider to be the first complete mt genome of *E. granulosus* genotype G1 and characterise all repeat regions in this genome.

## Methods

High molecular weight genomic DNA, extracted from protoscoleces from a single cyst of *E. granulosus* genotype G1 from a liver of a sheep from New South Wales in Australia using a conventional phenol:chloroform method [26], was sequenced using the PacBio Sequel [27] and BGISEQ-500 short-read sequencing [28] platforms employing established protocols. Sequence data obtained were assembled using a recently established common workflow language (CWL)-based pipeline [25]. To check the contiguity and sequencing depth of the assembly, the program Circlator v1.5.5 [29] was used to map corrected PacBio reads to the assembly; subsequently, short-read data were mapped using the program Bowtie2 v2.1.0 [30] and sorted using the program SAMtools v1.3.1 [31]. Aligned reads were inspected for any nucleotide inconsistencies using the program IGV v2.3.97 [32].

The mt genome was compared with a representative, published mt genome of *E. granulosus* genotype G1 (GenBank: AF297617; [21]), and its sequence deposited in the GenBank database under accession no. MK774655. Protein-encoding genes and rRNAs were annotated using an established bioinformatics pipeline [33]; tRNAs were identified using the same bioinformatic pipeline [33] and/or by a BLAST search [34] against an *E. granulosus* genotype G1 mt genome sequence available in GenBank (accession no. AB786664; [16]). The repeat region was characterised using the tandem repeat finder Dot2dot [35], and the secondary structures of non-coding regions were predicted using the RNAfold web server [36]. All annotations were curated manually.

## Results and discussion

**Fig. 1:**
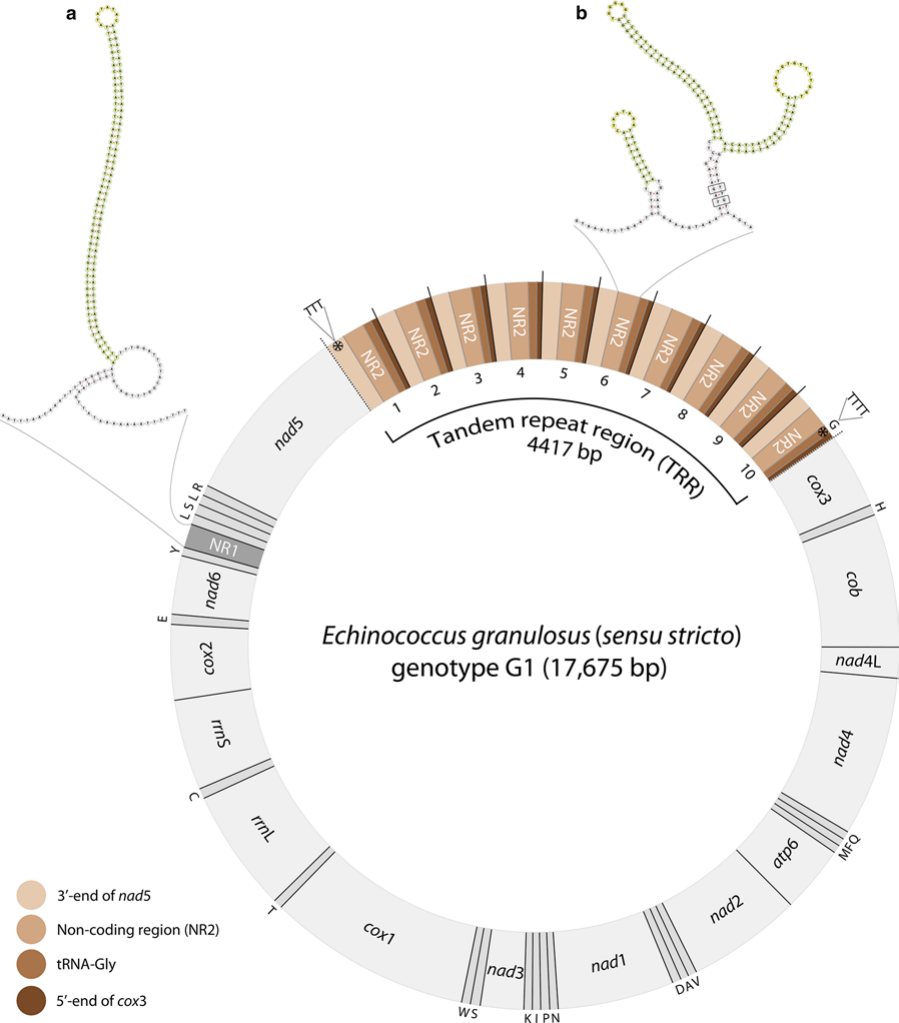
The complete mitochondrial genome of *Echinococcus granulosus* (*sensu stricto*) genotype G1. The 12 protein-encoding genes, 2 rRNAs and 21 tRNAs (except tRNA-Gly) are depicted in light grey; the non-coding region NR1 is in darker grey. Transfer RNAs are designated by one-letter amino acid abbreviations; gene designations follow Le et al. [83]. The tandem repeat region (in four shades of brown) spans 4417 bp and includes 10 repeat units. Each unit contains the 3′-end of *nad*5, the non-coding region NR2, tRNA-Gly (proposed pseudo-tRNAs in repeat units 1–9), 3 bp-intergenic region (not shown on figure) and 5′-end of *cox*3. Repeat units 2–9 are identical, whereas units 1 and 10 each have a 3–4 nucleotide insertion, marked by an asterisk. The TTT insertion occurs in repeat unit 1, at the 3′-end of *nad*5, the TTTT insertion occurs in repeat unit 10, in tRNA-Gly. Secondary structures of NR1 (**a**) and NR2 (**b**) are shown at the top; parts of these structures are predicted to have hair-pin loops with no mis-matches - depicted in green (stem) and yellow (loop); mis-matches are boxed

From totals of 2757 PacBio long-reads equating to 41 Mb, we assembled a complete mt genome for *E. granulosus* genotype G1 at an average sequencing depth of 2268, resulting in a contig of 17,675 bp, which is > 4 kb larger than all published mt genomes representing genotype G1 (~ 13,600 bp) [16, 21, 22], but with the same order of protein-coding genes (Fig. 1).

We succeeded in assembling a tandem repeat region (TRR) of 4417 bp; 1895 reads spanned this and flanking regions (≥ 1 kb, both 5′ and 3′). No length or sequence variation was detected among these reads, indicating a lack of polymorphism within or among protoscoleces within the sample from one cyst. The annotation of TRR revealed that it contains 10 tandemly repeated, near-identical repeat units: the first unit is 444 bp in length; units two to nine are identical in sequence, being 441 bp; and the tenth unit is 445 bp (Fig. 1). Each repeat unit within TRR contains 144–147 bp of the 3′-end of *nad*5, 184 bp of the non-coding region NR2, 63–67 bp of tRNA-Gly, a 3 bp-intergenic region and 47 bp of the start of the *cox*3 gene (Fig. 1). The tRNA in the tenth unit appears to be the only functional tRNA-Gly in the genome, but this suggestion requires experimental verification. In addition to characterising the 4417 bp intergenic region (TRR), we also succeeded in defining the complete sequence of the non-coding region NR1, which equated to a total of 183 bp (Fig. 1), the same length as estimated previously for *E. multilocularis* [37] but longer than the 87 bp [16] or 66 bp [21] recorded for *E. granulosus* genotype G1. Parts of NR1 and NR2 were predicted to fold into secondary stem-loop structures with possible roles in the regulation of replication and/or transcription of the mt genome (Fig. 1).

Sanger-sequencing and second-generation short-read sequencing, used previously to sequence mt genomes of *Echinococcus* [7, 16, 21–23, 37–39], are not suited to defining complex genomic regions, such as repeat elements. However, PacBio single-molecule real-time sequencing offers the long-read lengths to identify and characterise long, complicated repetitive regions [24], as achieved here. Other recent examples of success with PacBio sequencing include the resolution of unique, complex sequence tracts of ~ 4 kb and ~ 6.9 kb in the mt genomes of *Schistosoma bovis* [40] and *Paragonimus westermani* [41], respectively.

Published mt genomes of parasitic and free-living flatworms (e.g. [40–46]) are known to harbour non-coding regions of varying sizes, which may contain repeat elements. Two NCRs (66–875 bp) [47] appear to be characteristic of cestodes, in which repetitive elements are relatively common [47–55]. In *E. granulosus* genotype G1, the two NCRs are located between two tRNAs upstream of the *nad*5 gene (NR1) and between *nad*5 and tRNA-Gly (NR2), and have both been estimated at < 200 bp [16, 21]. Our results are consistent with the previous observations, in terms of the location of NR1 and NR2, and the length and sequence of NR2; however, the tandem replication of NR2 and its adjacent sequences are unique features not previously reported for mt genomes of cestodes.

In the future, the nature and extent of polymorphism in the tandem repeat region should be assessed by sequencing a large number of genotype G1 samples from individual cysts from distinct hosts and geographical locations using the PacBio approach, as the consistency of occurrence, size and sequence in this repeat region are presently unknown. It is possible that not all G1 isolates harbour tandem repeats, as they had not been observed in the published mitogenomes of G1 [16, 21, 22]. However, as the sequencing approaches used previously might not have been able to resolve complex regions, there is a question regarding the completeness of previously published mt genome sequences. In addition to the intra-genotypic variation, length and/or structural differences in TRR among different *Echinococcus* taxa should also be explored. The mt genome of genotype G3 is likely to harbour the tandem repeat region as well, as we detected but could not resolve the enigmatic region for 39 G3 samples using Sanger-based sequencing [56]. However, this might not be the case for genotypes G6 and G7, as Sanger-based sequencing defined, without complication, complete mt genomes (n = 94) for these genotypes in a recent study [7]. Taken together, these findings suggest that there is significant scope for studies of the nature and extent of variation in repeat regions within and among different *Echinococcus* species and genotypes, and their evolution.

We hypothesise that tandem repeats within genotype G1 might provide an evolutionary advantage over mt genomes with no such replications. Most mt genomes of animals are relatively small (typically 15–20 kb in size; [57, 58]), lack introns and have short intergenic regions (usually only a few bp; [59]) and are, thus, thought to be under selection for compactness (cf. [60]). Non-functional replications could be rare and would be expected to be eliminated relatively quickly due to the rapid rate of replication of compact mt genomes [60, 61]. It could be speculated that the existence of the tandem repeat region (TRR) within the mt genome of G1 overrides the selection for a small genome size and might provide an evolutionary advantage. A key element of this proposal could be the existence of replicated control regions (CRs) within TRR.

It is well established that mt genomes of animals contain a control region that initiates replication and transcription [62, 63]. Interestingly, there have been several reports of duplications of the control region in the mt genomes of various species of animals [64–68], which are thought to be advantageous, in terms of more efficient transcription and/or replication of mt genes [64, 65, 69]. As a working hypothesis, we propose that the 184 bp non-coding sequence (NR2) within each repeat unit of TRR is a putative control region of genotype G1 and, thus, the mt genome contains 10 identical copies of CR which might be beneficial, in terms of more efficient replication and/or transcription. Parts of this region appear to be capable of folding into secondary stem-loop structures (see Fig. 1), which, as suggested previously [37, 42, 44, 55], could be associated with mt genome replication in cestodes. This hypothesis warrants testing.

If the mt genome of *E. granulosus* genotype G1 did consistently contain 10 identical CRs, this might provide an advantage, in terms of cellular energy production, especially during life-cycle phases that require short-term bursts of energy in a micro-aerobic habitat. As an adaptation to this environment, it has been hypothesised that the parasite uses fermentative pathways to generate cellular energy, specifically lactic fermentation and malate dismutation [70–72]. While lactate is produced in the cytosol and excreted, mt fermentation of malate is known to occur in helminths [73] and is encoded in *Echinococcus* [74]. More effective mt replication and/or transcription mechanisms might compensate for the lower energy yield of fermentation [73] compared with aerobic respiration [75] and be under strong selective pressure. Efficient energy production would be particularly important during the phase in which eggs hatch, oncospheres activate and are then required to rapidly penetrate the intestinal wall of the intermediate host animal [76, 77]. The successful development of an *Echinococcus* cyst in an intermediate host is highly dependent on a rapid penetration of the oncosphere and immediate post-oncospheral establishment [77, 78]. Interestingly, genotype G1 has the broadest host range of all *Echinococcus* taxa [2]. Thus, it could be proposed that efficient energy production at the oncosphere stage might be one of the factors contributing to this genotype’s success at infecting a diverse range of host species. Another crucial phase requiring rapid energy production is during the development of protoscoleces into adult worms in the small intestine of the definitive host [77].

We suggest that the other non-coding region, NR1, might have an exclusive functional role in the replication of the mt genome. Several mechanisms, including rolling circle, strand-displacement, and strand-coupled replication, have been proposed for mt DNA in vertebrates and invertebrates [62]. Although the replication mechanisms in cestodes are not understood, the secondary structure of NR1 (see Fig. 1) seems to lend support to the strand-displacement mechanism being utilised (cf. [63]). According to this model, there are two distinct origins of replication, a CR containing the origin of leading-strand replication and another origin initiating lagging-strand replication, which is characterised by a stem-loop structure [63]. The 183 bp non-coding region assembled here, for the first time, for genotype G1 appears to assume a long stem-loop (Fig. 1), suggesting that, if the strand-displacement mechanism is utilised by the *Echinococcus* tapeworms, NR1 could be the initiation site for lagging-strand replication. The NR1 (183 bp) identified in *E. multilocularis* [37] is also predicted to fold into a long stem-loop of a similar size [44], suggesting that it has structural and functional significance in the mt genome. Future work might focus on exploring the roles of both NR1 and NR2 using 2D neutral agarose gel electrophoresis, Southern blot-hybridisation and electron microscopy techniques [79–82].

## Conclusions

Here, we report what we consider to be the first complete mt genome of *E. granulosus* genotype G1. We succeeded in defining an elusive tandem repeat region (4.4 kb), which consists of ten repeat units, each harbouring a 184 bp non-coding region and adjacent regions, a unique feature, not previously observed in mt genomes of cestodes. We also characterised a short non-coding region (183 bp; containing a long, inverted repeat) for the first time for genotype G1. The presence, size, sequence and function of tandem repeat regions in different isolates of genotype G1, and in other genotypes and species, remain to be studied. The discovery here of “new” repeat elements in the mt genome of G1 raises a question about the completeness of some published genomes of taeniidae assembled previously from conventional or short-read sequence data sets. The present study shows that PacBio sequencing overcomes the challenges associated with the assembly of repeat elements in genomes and indicates its benefits for investigating the genomes of cestodes and other parasites.

## Data Availability

The data generated and analysed during the present study are available in the GenBank database under the accession no. MK774655. Raw data are available in the CNSA database under the accession no. CNP0000438.

## Abbreviations

WHO: World Health Organization
NTD: neglected tropical disease
mt: mitochondrial
bp: base pair or base pairs
kb: kilobase or kilobases
Mb: megabase or megabases
PCR: polymerase chain reaction
tRNA: transfer RNA
rRNA: ribosomal RNA
Leu: Leucine
Tyr: Tyrosine
Gly: Glycine
*cox*3: cytochrome *c* oxidase subunit 3
*nad*5: NADH dehydrogenase subunit 5
TRR: tandem repeat region
NCR: non-coding region
NR1: non-coding region 1
NR2: non-coding region 2
CR: control region
CWL: common workflow language
2D: two-dimensional
